# Supplementary material for: Inhibitory Effect of Quercetin on Oxidative Endogen Enzymes: A Focus on Putative Binding Modes
Source: Int J Mol Sci. 2023 Oct 20;24(20):15391. doi: 10.3390/ijms242015391 (PMC10607112; doi:10.3390/ijms242015391)
Supplement: Supplementary file 1 [file ijms-24-15391-s001.zip › ijms-2659855-supplementary.pdf]

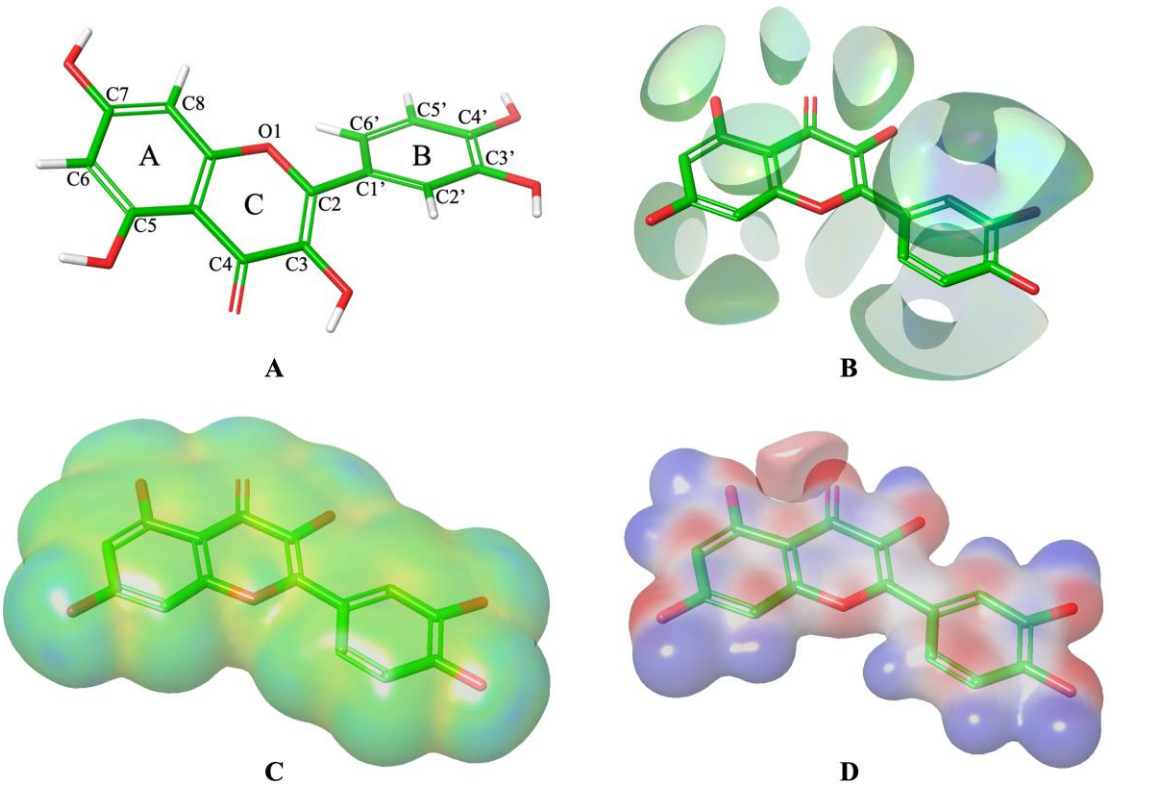

Figure S1. Optimized structure of Que (A), ALIE mapped molecular surface of Que (A), ED mapped molecular surface of Que (B), and ESP (C) mapped molecular surface of Que: red signifies electron-rich regions and blue indicates electron-poor zones.

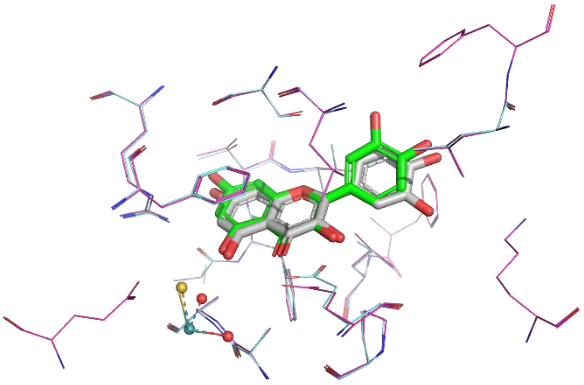

Figure S2. Superimposition of Que derived from the docking studies on XO (green sticks) over the native Que (grey sticks). Residues belonging to the binding sites are represented in lines, while the dioxothiomolybdenum(VI) ion is represented in small spheres.

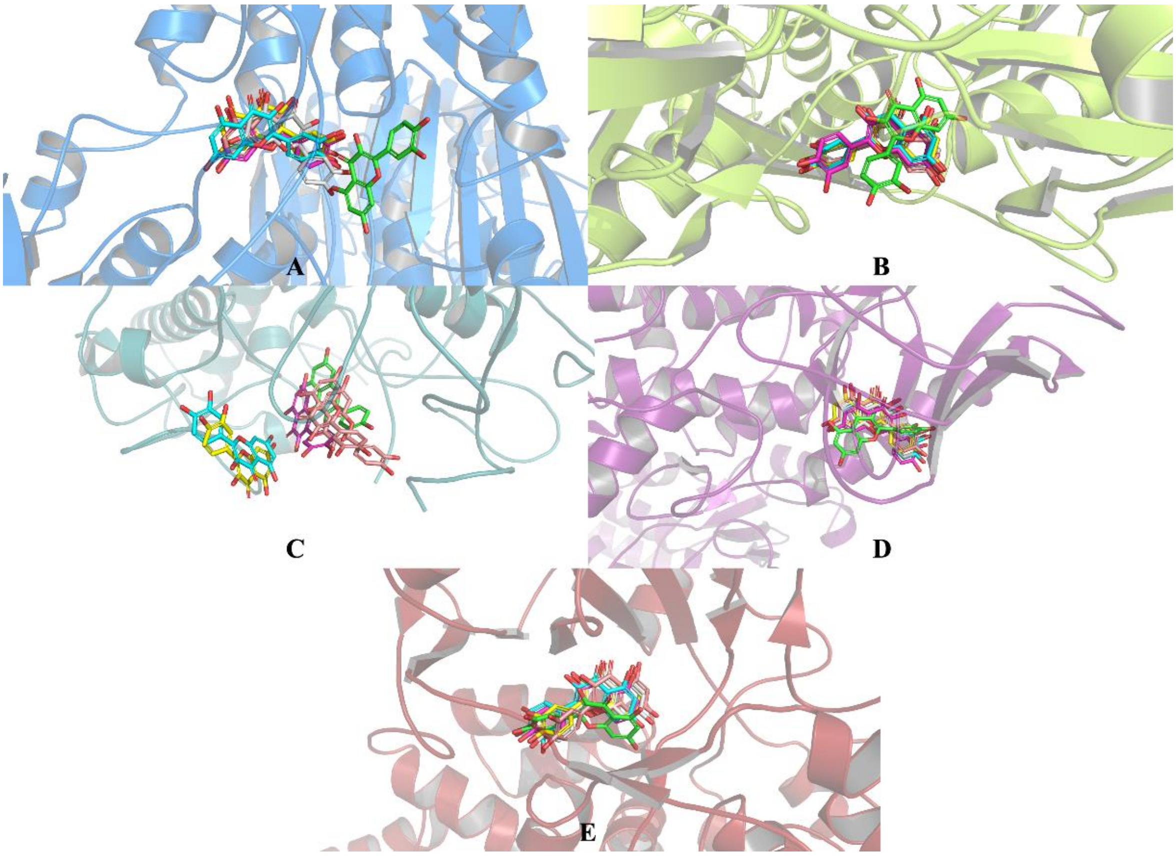

Figure S3. Results of the cluster analysis conducted on the MD trajectories of NOX (A), XO (B), MPO (C), 5-LOX (D), and MAO-A (E) aligned on the reference pose derived from docking studies on each enzyme. The chemical structures are colored as follows: reference docking pose (green), cluster 1 (cyan), cluster 2 (magenta), cluster 3 (yellow), cluster 4 (pink), and cluster 5 (grey).

Table S1. Que's calculated coordinates derived from DFT calculations: final geometry (A), bond length (B), bond angles (C), and torsional angles (D).

**(A) Final geometry**			
**Atom**	**X**	**Y**	**Z**
C1	−0.755	0.956	−0.789
C2	0.033	−0.029	−0.280
C3	−2.222	0.871	−0.808
C4	−2.790	−0.344	−0.247
O5	−0.569	−1.154	0.239
C6	−1.912	−1.319	0.260
C7	−4.182	−0.624	−0.171
C8	1.492	−0.085	−0.192
C9	−2.355	−2.518	0.819
C10	−4.635	−1.812	0.382
C11	2.301	0.960	−0.679
C12	3.683	0.906	−0.594
O13	−2.833	1.828	−1.295
C14	−3.722	−2.753	0.874
C15	4.306	−0.212	−0.009
C16	2.124	−1.194	0.387

O17	−0.282	2.104	−1.325
C18	3.510	−1.247	0.473
O19	−5.037	0.303	−0.653
O20	4.496	1.899	−1.054
O21	5.666	−0.214	0.049
O22	−4.251	−3.889	1.397
H23	−1.630	−3.230	1.191
H24	−5.694	−2.032	0.443
H25	1.841	1.829	−1.130
H26	1.535	−2.013	0.774
H27	−1.104	2.574	−1.586
H28	3.989	−2.112	0.925
H29	−5.940	−0.014	−0.539
H30	3.949	2.597	−1.429
H31	5.959	−1.030	0.467
H32	−3.543	−4.465	1.704

**(B) Bond length**			
**Atoms**			**Å**
C1	-C2	-C2	1.360
C1	-C3	-C3	1.470
C1	-O17	-O17	1.352
C2	-O5	-O5	1.378
C2	-C8	-C8	1.462
C3	-C4	-C4	1.454
C3	-O13	-O13	1.235
C4	-C6	-C6	1.407
C4	-C7	-C7	1.422
O5	-C6	-C6	1.353
C6	-C9	-C9	1.395
C7	-C10	-C10	1.386
C7	-O19	-O19	1.350
C8	-C11	-C11	1.409
C8	-C16	-C16	1.402
C9	-C14	-C14	1.388
C9	-H23	-H23	1.083
C10	-C14	-C14	1.401
C10	-H24	-H24	1.084
C11	-C12	-C12	1.386
C11	-H25	-H25	1.081
C12	-C15	-C15	1.407
C12	-O20	-O20	1.363
C14	-O22	-O22	1.358

C15	-C18	-C18	1.392
C15	-O21	-O21	1.361
C16	-C18	-C18	1.390
C16	-H26	-H26	1.080
O17	-H27	-H27	0.983
C18	-H28	-H28	1.086
O19	-H29	-H29	0.963
O20	-H30	-H30	0.963
O21	-H31	-H31	0.963
O22	-H32	-H32	0.963

**(C) Bond angles**			
**Atoms**			°
C3	-C1	-C2	122.808
O17	-C1	-C2	124.019
O17	-C1	-C3	113.173
O5	-C2	-C1	118.610
C8	-C2	-C1	128.966
C8	-C2	-O5	112.424
C4	-C3	-C1	115.664
O13	-C3	-C1	117.003
O13	-C3	-C4	127.334
C6	-C4	-C3	118.339
C7	-C4	-C3	124.582
C7	-C4	-C6	117.078
C6	-O5	-C2	122.603
O5	-C6	-C4	121.976
C9	-C6	-C4	122.800
C9	-C6	-O5	115.224
C10	-C7	-C4	120.636
O19	-C7	-C4	117.758
O19	-C7	-C10	121.605
C11	-C8	-C2	121.540
C16	-C8	-C2	120.369
C16	-C8	-C11	118.091
C14	-C9	-C6	118.346
H23	-C9	-C6	119.361
H23	-C9	-C14	122.293
C14	-C10	-C7	120.231
H24	-C10	-C7	121.052
H24	-C10	-C14	118.717
C12	-C11	-C8	121.477
H25	-C11	-C8	119.755

H25	-C11	-C12	118.768
C15	-C12	-C11	119.902
O20	-C12	-C11	123.033
O20	-C12	-C15	117.064
C10	-C14	-C9	120.908
O22	-C14	-C9	122.763
O22	-C14	-C10	116.328
C18	-C15	-C12	118.782
O21	-C15	-C12	117.483
O21	-C15	-C18	123.735
C18	-C16	-C8	120.377
H26	-C16	-C8	120.102
H26	-C16	-C18	119.521
H27	-O17	-C1	102.627
C16	-C18	-C15	121.371
H28	-C18	-C15	118.945
H28	-C18	-C16	119.684
H29	-O19	-C7	108.983
H30	-O20	-C12	108.691
H31	-O21	-C15	108.905
H32	-O22	-C14	109.665

**(D) Torsional angles**				
**Atoms**				°
C1	-C2	-O5	-C6	0.167
C1	-C2	-C8	-C11	−0.691
C1	-C2	-C8	-C16	179.288
C1	-C3	-C4	-C6	0.072
C1	-C3	-C4	-C7	−179.927
C2	-C1	-C3	-C4	−0.133
C2	-C1	-C3	-O13	179.917
C2	-C1	-O17	-H27	−179.959
C2	-O5	-C6	-C4	−0.231
C2	-O5	-C6	-C9	179.870
C2	-C8	-C11	-C12	179.963
C2	-C8	-C11	-H25	0.050
C2	-C8	-C16	-C18	−179.944
C2	-C8	-C16	-H26	−0.129
C3	-C1	-C2	-O5	0.020
C3	-C1	-C2	-C8	−179.942
C3	-C1	-O17	-H27	0.036
C3	-C4	-C6	-O5	0.101
C3	-C4	-C6	-C9	179.992

C3	-C4	-C7	-C10	179.998
C3	-C4	-C7	-O19	−0.007
C4	-C3	-C1	-O17	179.871
C4	-C6	-C9	-C14	0.034
C4	-C6	-C9	-H23	179.916
C4	-C7	-C10	-C14	−0.014
C4	-C7	-C10	-H24	−179.979
C4	-C7	-O19	-H29	179.965
O5	-C2	-C1	-O17	−179.985
O5	-C2	-C8	-C11	179.346
O5	-C2	-C8	-C16	−0.675
O5	-C6	-C4	-C7	−179.900
O5	-C6	-C9	-C14	179.931
O5	-C6	-C9	-H23	−0.186
C6	-C4	-C3	-O13	−179.984
C6	-C4	-C7	-C10	−0.001
C6	-C4	-C7	-O19	179.994
C6	-O5	-C2	-C8	−179.866
C6	-C9	-C14	-C10	−0.049
C6	-C9	-C14	-O22	179.947
C7	-C4	-C3	-O13	0.017
C7	-C4	-C6	-C9	−0.009
C7	-C10	-C14	-C9	0.040
C7	-C10	-C14	-O22	−179.956
C8	-C2	-C1	-O17	0.053
C8	-C11	-C12	-C15	−0.024
C8	-C11	-C12	-O20	−179.951
C8	-C16	-C18	-C15	−0.015
C8	-C16	-C18	-H28	179.931
C9	-C14	-C10	-H24	−179.994
C9	-C14	-O22	-H32	−0.008
C10	-C7	-O19	-H29	−0.040
C10	-C14	-C9	-H23	−179.928
C10	-C14	-O22	-H32	179.988
C11	-C8	-C16	-C18	0.036
C11	-C8	-C16	-H26	179.851
C11	-C12	-C15	-C18	0.044
C11	-C12	-C15	-O21	−179.994
C11	-C12	-O20	-H30	−0.273
C12	-C11	-C8	-C16	−0.016
C12	-C15	-C18	-C16	−0.025
C12	-C15	-C18	-H28	−179.971
C12	-C15	-O21	-H31	−179.916

O13	-C3	-C1	-O17	−0.078
C14	-C10	-C7	-O19	179.991
C15	-C12	-C11	-H25	179.891
C15	-C12	-O20	-H30	179.798
C15	-C18	-C16	-H26	−179.831
C16	-C8	-C11	-H25	−179.930
C16	-C18	-C15	-O21	−179.984
C18	-C15	-C12	-O20	179.975
C18	-C15	-O21	-H31	0.043
O19	-C7	-C10	-H24	0.026
O20	-C12	-C11	-H25	−0.036
O20	-C12	-C15	-O21	−0.063
O21	-C15	-C18	-H28	0.070
O22	-C14	-C9	-H23	0.068
O22	-C14	-C10	-H24	0.010
H26	-C16	-C18	-H28	0.115

Table S2. Natural population analysis derived from NBO analysis.

Atom N.	Charge	Valence	Rydberg	Total
C1	0.208	3.767	0.025	5.792
C2	0.292	3.683	0.025	5.708
C3	0.465	3.500	0.035	5.535
C4	-0.250	4.230	0.020	6.250
O5	-0.495	6.474	0.020	8.495
C6	0.391	3.587	0.022	5.609
C7	0.409	3.566	0.025	5.591
C8	-0.106	4.087	0.018	6.106
C9	-0.354	4.336	0.018	6.354
C10	-0.357	4.337	0.020	6.357
C11	-0.235	4.219	0.016	6.235
C12	0.277	3.699	0.023	5.723
O13	-0.617	6.601	0.016	8.617
C14	0.368	3.610	0.022	5.632
C15	0.290	3.687	0.024	5.710
C16	-0.176	4.158	0.018	6.176
O17	-0.695	6.678	0.017	8.695
C18	-0.272	4.254	0.018	6.272
O19	-0.636	6.621	0.015	8.636
O20	-0.667	6.653	0.014	8.667
O21	-0.659	6.645	0.014	8.659
O22	-0.667	6.654	0.013	8.667
H23	0.220	0.777	0.003	0.780
H24	0.220	0.778	0.002	0.780

H25	0.236	0.761	0.003	0.764
H26	0.219	0.778	0.003	0.781
H27	0.501	0.493	0.006	0.499
H28	0.203	0.795	0.002	0.797
H29	0.470	0.526	0.004	0.530
H30	0.472	0.524	0.004	0.528
H31	0.469	0.526	0.004	0.531
H32	0.475	0.521	0.004	0.525

Table S3. Second order perturbation analyis of Fock matrix in NBO basis. E(2) is expressed in kcal/mol, while E(NL)-E(L) and F(L,NL) are expressed in atomic units.

Donor (L) NBO	Acceptor (NL) NBO	E(2)	E(NL)-E(L)	F(L,NL)
23. LP (1) O5	79. BD*(1) C1-C2	4.230	1.080	0.060
23. LP (1) O5	82. BD*(1) C1-O17	0.510	0.860	0.019
23. LP (1) O5	84. BD*(1) C2-C8	1.300	0.960	0.032
23. LP (1) O5	88. BD*(1) C4-C6	6.000	1.020	0.070
23. LP (1) O5	92. BD*(1) C6-C9	1.290	1.030	0.033
23. LP (1) O5	137. RY (1) C2	1.490	1.740	0.045
23. LP (1) O5	139. RY (3) C2	0.590	2.560	0.035
23. LP (1) O5	203. RY (1) C6	2.560	1.910	0.062
24. LP (2) O5	80. BD*(2) C1-C2	26.520	0.380	0.089
24. LP (2) O5	89. BD*(2) C4-C6	35.180	0.360	0.100
24. LP (2) O5	140. RY (4) C2	1.540	1.500	0.043
24. LP (2) O5	141. RY (5) C2	0.830	1.730	0.034
24. LP (2) O5	205. RY (3) C6	2.310	1.860	0.058
25. LP (1) O13	81. BD*(1) C1-C3	1.360	0.990	0.033
25. LP (1) O13	85. BD*(1) C3-C4	0.880	1.020	0.027
25. LP (1) O13	115. BD*(1) O17-H27	0.670	0.950	0.023
25. LP (1) O13	154. RY (1) C3	8.840	1.630	0.107
25. LP (1) O13	167. RY (14) C3	0.550	7.510	0.057
26. LP (2) O13	79. BD*(1) C1-C2	0.510	0.840	0.019
26. LP (2) O13	81. BD*(1) C1-C3	18.330	0.700	0.101
26. LP (2) O13	85. BD*(1) C3-C4	19.230	0.730	0.105
26. LP (2) O13	115. BD*(1) O17-H27	6.640	0.660	0.059
26. LP (2) O13	154. RY (1) C3	0.560	1.350	0.025
26. LP (2) O13	155. RY (2) C3	2.040	1.990	0.057
26. LP (2) O13	157. RY (4) C3	1.840	2.390	0.059
27. LP (1) O17	81. BD*(1) C1-C3	4.300	0.970	0.058
27. LP (1) O17	106. BD*(1) C11-H25	2.040	0.990	0.040
27. LP (1) O17	121. RY (1) C1	2.280	1.660	0.055
27. LP (1) O17	124. RY (4) C1	0.510	1.950	0.028
27. LP (1) O17	512. RY (1) H27	0.690	2.270	0.035
28. LP (2) O17	80. BD*(2) C1-C2	33.920	0.340	0.096

28. LP (2) O17	123. RY (3) C1	2.580	1.840	0.061
28. LP (2) O17	513. RY (2) H27	1.520	1.860	0.047
29. LP (1) O19	90. BD*(1) C4-C7	0.880	1.030	0.027
29. LP (1) O19	93. BD*(1) C7-C10	5.300	1.080	0.067
29. LP (1) O19	220. RY (1) C7	2.210	1.790	0.056
29. LP (1) O19	222. RY (3) C7	0.690	2.190	0.035
29. LP (1) O19	525. RY (2) H29	1.390	2.280	0.050
30. LP (2) O19	94. BD*(2) C7-C10	37.670	0.340	0.101
30. LP (2) O19	221. RY (2) C7	2.440	1.880	0.060
30. LP (2) O19	524. RY (1) H29	2.000	1.860	0.054
31. LP (1) O20	104. BD*(1) C11-C12	5.330	1.100	0.068
31. LP (1) O20	107. BD*(1) C12-C15	0.970	1.050	0.029
31. LP (1) O20	302. RY (1) C12	1.990	1.720	0.052
31. LP (1) O20	305. RY (4) C12	0.770	2.610	0.040
31. LP (1) O20	531. RY (2) H30	1.340	2.300	0.050
32. LP (2) O20	105. BD*(2) C11-C12	30.830	0.350	0.092
32. LP (2) O20	304. RY (3) C12	2.220	1.880	0.058
32. LP (2) O20	530. RY (1) H30	1.990	1.860	0.054
33. LP (1) O21	107. BD*(1) C12-C15	0.980	1.060	0.029
33. LP (1) O21	110. BD*(1) C15-C18	5.600	1.090	0.070
33. LP (1) O21	353. RY (1) C15	1.900	1.720	0.051
33. LP (1) O21	356. RY (4) C15	0.680	2.140	0.034
33. LP (1) O21	537. RY (2) H31	1.430	2.200	0.050
34. LP (2) O21	111. BD*(2) C15-C18	32.370	0.350	0.095
34. LP (2) O21	355. RY (3) C15	2.230	1.880	0.058
34. LP (2) O21	536. RY (1) H31	2.030	1.860	0.055
35. LP (1) O22	99. BD*(1) C9-C14	5.570	1.100	0.070
35. LP (1) O22	102. BD*(1) C10-C14	0.640	1.080	0.023
35. LP (1) O22	336. RY (1) C14	2.410	1.680	0.057
35. LP (1) O22	339. RY (4) C14	0.540	2.350	0.032
35. LP (1) O22	543. RY (2) H32	1.350	2.320	0.050
36. LP (2) O22	100. BD*(2) C9-C14	34.990	0.350	0.099
36. LP (2) O22	338. RY (3) C14	2.230	1.890	0.058
36. LP (2) O22	542. RY (1) H32	1.930	1.860	0.054
37. BD (1) C1-C2	81. BD*(1) C1-C3	2.110	1.100	0.043
37. BD (1) C1-C2	82. BD*(1) C1-O17	1.050	1.030	0.029
37. BD (1) C1-C2	84. BD*(1) C2-C8	3.400	1.120	0.055
37. BD (1) C1-C2	86. BD*(1) C3-O13	1.110	1.230	0.033
37. BD (1) C1-C2	97. BD*(1) C8-C16	1.450	1.210	0.037
37. BD (1) C1-C2	115. BD*(1) O17-H27	1.200	1.060	0.032
37. BD (1) C1-C2	157. RY (4) C3	0.780	2.780	0.041
37. BD (1) C1-C2	186. RY (1) O5	0.560	1.520	0.026
37. BD (1) C1-C2	237. RY (2) C8	2.060	2.100	0.059

38. BD (2) C1-C2	87. BD*(2) C3-O13	21.780	0.300	0.072
38. BD (2) C1-C2	98. BD*(2) C8-C16	13.440	0.320	0.059
38. BD (2) C1-C2	188. RY (3) O5	1.000	0.970	0.028
38. BD (2) C1-C2	239. RY (4) C8	0.960	1.090	0.029
38. BD (2) C1-C2	386. RY (1) O17	1.750	0.770	0.033
39. BD (1) C1-C3	79. BD*(1) C1-C2	2.420	1.180	0.048
39. BD (1) C1-C3	83. BD*(1) C2-O5	0.970	0.930	0.027
39. BD (1) C1-C3	84. BD*(1) C2-C8	3.060	1.060	0.051
39. BD (1) C1-C3	85. BD*(1) C3-C4	0.720	1.060	0.025
39. BD (1) C1-C3	90. BD*(1) C4-C7	2.410	1.100	0.046
39. BD (1) C1-C3	137. RY (1) C2	0.810	1.840	0.035
39. BD (1) C1-C3	171. RY (2) C4	1.470	2.080	0.049
39. BD (1) C1-C3	319. RY (1) O13	0.800	1.390	0.030
40. BD (1) C1-O17	79. BD*(1) C1-C2	1.280	1.390	0.038
40. BD (1) C1-O17	83. BD*(1) C2-O5	2.070	1.130	0.043
40. BD (1) C1-O17	85. BD*(1) C3-C4	1.050	1.270	0.033
41. BD (1) C2-O5	82. BD*(1) C1-O17	2.360	1.180	0.047
41. BD (1) C2-O5	92. BD*(1) C6-C9	1.680	1.350	0.042
41. BD (1) C2-O5	96. BD*(1) C8-C11	1.430	1.360	0.039
41. BD (1) C2-O5	204. RY (2) C6	1.860	2.590	0.062
41. BD (1) C2-O5	237. RY (2) C8	1.030	2.250	0.043
42. BD (1) C2-C8	79. BD*(1) C1-C2	2.900	1.170	0.052
42. BD (1) C2-C8	81. BD*(1) C1-C3	1.830	1.030	0.039
42. BD (1) C2-C8	91. BD*(1) O5-C6	2.450	0.950	0.043
42. BD (1) C2-C8	96. BD*(1) C8-C11	1.860	1.140	0.041
42. BD (1) C2-C8	97. BD*(1) C8-C16	2.110	1.140	0.044
42. BD (1) C2-C8	104. BD*(1) C11-C12	1.430	1.160	0.036
42. BD (1) C2-C8	113. BD*(1) C16-C18	1.520	1.160	0.038
42. BD (1) C2-C8	121. RY (1) C1	1.070	1.730	0.038
42. BD (1) C2-C8	186. RY (1) O5	0.630	1.450	0.027
42. BD (1) C2-C8	287. RY (2) C11	0.820	1.740	0.034
42. BD (1) C2-C8	370. RY (1) C16	1.230	1.760	0.042
43. BD (1) C3-C4	81. BD*(1) C1-C3	0.610	1.040	0.022
43. BD (1) C3-C4	82. BD*(1) C1-O17	1.890	0.960	0.038
43. BD (1) C3-C4	86. BD*(1) C3-O13	1.330	1.170	0.035
43. BD (1) C3-C4	88. BD*(1) C4-C6	1.720	1.120	0.039
43. BD (1) C3-C4	90. BD*(1) C4-C7	2.010	1.100	0.042
43. BD (1) C3-C4	91. BD*(1) O5-C6	0.550	0.950	0.020
43. BD (1) C3-C4	92. BD*(1) C6-C9	2.360	1.140	0.046
43. BD (1) C3-C4	93. BD*(1) C7-C10	1.640	1.150	0.039
43. BD (1) C3-C4	122. RY (2) C1	0.500	2.250	0.030
43. BD (1) C3-C4	203. RY (1) C6	0.720	2.010	0.034
43. BD (1) C3-C4	220. RY (1) C7	1.270	1.860	0.043

43. BD (1) C3-C4	319. RY (1) O13	0.750	1.390	0.029
44. BD (1) C3-O13	79. BD*(1) C1-C2	0.950	1.460	0.033
44. BD (1) C3-O13	81. BD*(1) C1-C3	0.850	1.320	0.030
44. BD (1) C3-O13	85. BD*(1) C3-C4	1.940	1.340	0.046
44. BD (1) C3-O13	88. BD*(1) C4-C6	0.770	1.400	0.029
44. BD (1) C3-O13	122. RY (2) C1	1.180	2.530	0.049
44. BD (1) C3-O13	170. RY (1) C4	1.420	2.350	0.052
45. BD (2) C3-O13	80. BD*(2) C1-C2	6.500	0.390	0.045
45. BD (2) C3-O13	89. BD*(2) C4-C6	5.410	0.370	0.040
46. BD (1) C4-C6	85. BD*(1) C3-C4	2.030	1.090	0.042
46. BD (1) C4-C6	86. BD*(1) C3-O13	2.630	1.200	0.050
46. BD (1) C4-C6	90. BD*(1) C4-C7	2.210	1.130	0.045
46. BD (1) C4-C6	92. BD*(1) C6-C9	2.860	1.160	0.051
46. BD (1) C4-C6	95. BD*(1) C7-O19	2.680	0.980	0.046
46. BD (1) C4-C6	101. BD*(1) C9-H23	1.800	1.030	0.039
46. BD (1) C4-C6	157. RY (4) C3	0.810	2.750	0.042
46. BD (1) C4-C6	186. RY (1) O5	0.870	1.490	0.032
46. BD (1) C4-C6	220. RY (1) C7	0.670	1.890	0.032
46. BD (1) C4-C6	252. RY (1) C9	1.010	1.890	0.039
46. BD (1) C4-C6	253. RY (2) C9	0.830	1.710	0.034
47. BD (2) C4-C6	87. BD*(2) C3-O13	28.660	0.290	0.081
47. BD (2) C4-C6	89. BD*(2) C4-C6	3.550	0.280	0.028
47. BD (2) C4-C6	94. BD*(2) C7-C10	24.570	0.280	0.075
47. BD (2) C4-C6	100. BD*(2) C9-C14	12.650	0.280	0.053
47. BD (2) C4-C6	156. RY (3) C3	0.600	1.860	0.030
47. BD (2) C4-C6	159. RY (6) C3	0.590	1.650	0.028
47. BD (2) C4-C6	188. RY (3) O5	1.200	0.950	0.030
47. BD (2) C4-C6	225. RY (6) C7	1.060	1.550	0.036
47. BD (2) C4-C6	254. RY (3) C9	1.790	0.770	0.033
48. BD (1) C4-C7	81. BD*(1) C1-C3	0.940	1.050	0.028
48. BD (1) C4-C7	85. BD*(1) C3-C4	2.460	1.080	0.046
48. BD (1) C4-C7	88. BD*(1) C4-C6	2.200	1.130	0.045
48. BD (1) C4-C7	91. BD*(1) O5-C6	3.480	0.970	0.052
48. BD (1) C4-C7	93. BD*(1) C7-C10	2.600	1.160	0.049
48. BD (1) C4-C7	95. BD*(1) C7-O19	0.510	0.970	0.020
48. BD (1) C4-C7	103. BD*(1) C10-H24	2.060	1.020	0.041
48. BD (1) C4-C7	117. BD*(1) O19-H29	1.910	1.000	0.039
48. BD (1) C4-C7	154. RY (1) C3	0.870	1.700	0.034
48. BD (1) C4-C7	158. RY (5) C3	0.790	3.120	0.044
48. BD (1) C4-C7	204. RY (2) C6	0.990	2.390	0.043
48. BD (1) C4-C7	269. RY (1) C10	0.910	1.770	0.036
48. BD (1) C4-C7	270. RY (2) C10	0.560	1.560	0.026
48. BD (1) C4-C7	420. RY (1) O19	0.500	1.570	0.025

49. BD (1) O5-C6	84. BD*(1) C2-C8	1.170	1.300	0.035
49. BD (1) O5-C6	90. BD*(1) C4-C7	0.990	1.340	0.033
49. BD (1) O5-C6	99. BD*(1) C9-C14	0.870	1.400	0.031
49. BD (1) O5-C6	138. RY (2) C2	1.010	2.540	0.045
49. BD (1) O5-C6	252. RY (1) C9	0.670	2.100	0.033
50. BD (1) C6-C9	83. BD*(1) C2-O5	2.580	0.960	0.044
50. BD (1) C6-C9	85. BD*(1) C3-C4	1.960	1.090	0.041
50. BD (1) C6-C9	88. BD*(1) C4-C6	2.750	1.150	0.050
50. BD (1) C6-C9	99. BD*(1) C9-C14	1.980	1.180	0.043
50. BD (1) C6-C9	101. BD*(1) C9-H23	1.270	1.030	0.032
50. BD (1) C6-C9	109. BD*(1) C14-O22	3.780	0.960	0.054
50. BD (1) C6-C9	171. RY (2) C4	1.070	2.110	0.042
50. BD (1) C6-C9	186. RY (1) O5	1.080	1.490	0.036
50. BD (1) C6-C9	337. RY (2) C14	1.650	2.210	0.054
51. BD (1) C7-C10	85. BD*(1) C3-C4	2.200	1.100	0.044
51. BD (1) C7-C10	90. BD*(1) C4-C7	3.050	1.130	0.052
51. BD (1) C7-C10	102. BD*(1) C10-C14	1.890	1.170	0.042
51. BD (1) C7-C10	103. BD*(1) C10-H24	1.120	1.040	0.031
51. BD (1) C7-C10	109. BD*(1) C14-O22	2.610	0.970	0.045
51. BD (1) C7-C10	170. RY (1) C4	1.240	2.100	0.046
51. BD (1) C7-C10	336. RY (1) C14	1.730	1.770	0.049
52. BD (2) C7-C10	89. BD*(2) C4-C6	12.550	0.280	0.053
52. BD (2) C7-C10	94. BD*(2) C7-C10	2.160	0.290	0.022
52. BD (2) C7-C10	100. BD*(2) C9-C14	27.630	0.280	0.079
52. BD (2) C7-C10	172. RY (3) C4	1.280	0.840	0.029
52. BD (2) C7-C10	340. RY (5) C14	0.670	1.910	0.032
52. BD (2) C7-C10	343. RY (8) C14	0.660	0.780	0.020
52. BD (2) C7-C10	421. RY (2) O19	1.390	0.870	0.031
53. BD (1) C7-O19	88. BD*(1) C4-C6	1.250	1.350	0.037
53. BD (1) C7-O19	90. BD*(1) C4-C7	0.550	1.330	0.024
53. BD (1) C7-O19	93. BD*(1) C7-C10	0.670	1.380	0.027
53. BD (1) C7-O19	102. BD*(1) C10-C14	0.820	1.370	0.030
53. BD (1) C7-O19	170. RY (1) C4	0.990	2.300	0.043
54. BD (1) C8-C11	83. BD*(1) C2-O5	2.140	0.920	0.040
54. BD (1) C8-C11	84. BD*(1) C2-C8	1.620	1.050	0.037
54. BD (1) C8-C11	97. BD*(1) C8-C16	3.000	1.130	0.052
54. BD (1) C8-C11	104. BD*(1) C11-C12	2.090	1.160	0.044
54. BD (1) C8-C11	106. BD*(1) C11-H25	1.460	1.040	0.035
54. BD (1) C8-C11	108. BD*(1) C12-O20	3.180	0.940	0.049
54. BD (1) C8-C11	114. BD*(1) C16-H26	2.260	1.040	0.043
54. BD (1) C8-C11	137. RY (1) C2	1.640	1.830	0.049
54. BD (1) C8-C11	303. RY (2) C12	1.540	3.510	0.066
54. BD (1) C8-C11	370. RY (1) C16	0.960	1.750	0.037

55. BD (1) C8-C16	79. BD*(1) C1-C2	1.580	1.170	0.038
55. BD (1) C8-C16	83. BD*(1) C2-O5	0.620	0.920	0.021
55. BD (1) C8-C16	84. BD*(1) C2-C8	1.780	1.050	0.039
55. BD (1) C8-C16	96. BD*(1) C8-C11	2.900	1.130	0.051
55. BD (1) C8-C16	106. BD*(1) C11-H25	2.290	1.040	0.044
55. BD (1) C8-C16	113. BD*(1) C16-C18	2.260	1.160	0.046
55. BD (1) C8-C16	114. BD*(1) C16-H26	1.060	1.040	0.030
55. BD (1) C8-C16	116. BD*(1) C18-H28	2.170	1.010	0.042
55. BD (1) C8-C16	137. RY (1) C2	1.530	1.830	0.047
55. BD (1) C8-C16	138. RY (2) C2	0.520	2.280	0.031
55. BD (1) C8-C16	286. RY (1) C11	0.710	1.720	0.031
55. BD (1) C8-C16	287. RY (2) C11	0.540	1.740	0.027
55. BD (1) C8-C16	403. RY (1) C18	0.710	1.750	0.032
55. BD (1) C8-C16	404. RY (2) C18	1.200	1.810	0.042
55. BD (1) C8-C16	506. RY (1) H26	0.820	1.420	0.030
56. BD (2) C8-C16	80. BD*(2) C1-C2	19.260	0.270	0.065
56. BD (2) C8-C16	105. BD*(2) C11-C12	19.480	0.280	0.066
56. BD (2) C8-C16	111. BD*(2) C15-C18	19.880	0.270	0.065
56. BD (2) C8-C16	140. RY (4) C2	0.640	1.400	0.027
56. BD (2) C8-C16	141. RY (5) C2	0.680	1.630	0.030
56. BD (2) C8-C16	288. RY (3) C11	1.940	1.090	0.041
56. BD (2) C8-C16	405. RY (3) C18	1.540	1.300	0.040
57. BD (1) C9-C14	91. BD*(1) O5-C6	2.950	0.990	0.048
57. BD (1) C9-C14	92. BD*(1) C6-C9	2.050	1.170	0.044
57. BD (1) C9-C14	101. BD*(1) C9-H23	1.190	1.040	0.031
57. BD (1) C9-C14	102. BD*(1) C10-C14	2.700	1.170	0.050
57. BD (1) C9-C14	103. BD*(1) C10-H24	1.860	1.050	0.039
57. BD (1) C9-C14	203. RY (1) C6	1.430	2.050	0.048
57. BD (1) C9-C14	204. RY (2) C6	0.720	2.410	0.037
57. BD (1) C9-C14	269. RY (1) C10	0.790	1.790	0.034
57. BD (1) C9-C14	471. RY (1) O22	0.570	1.290	0.024
58. BD (2) C9-C14	89. BD*(2) C4-C6	28.900	0.280	0.081
58. BD (2) C9-C14	94. BD*(2) C7-C10	12.220	0.290	0.053
58. BD (2) C9-C14	100. BD*(2) C9-C14	2.380	0.280	0.023
58. BD (2) C9-C14	207. RY (5) C6	0.920	1.840	0.037
58. BD (2) C9-C14	272. RY (4) C10	1.890	0.750	0.034
58. BD (2) C9-C14	472. RY (2) O22	1.340	0.920	0.031
59. BD (1) C9-H23	88. BD*(1) C4-C6	3.500	1.030	0.054
59. BD (1) C9-H23	91. BD*(1) O5-C6	1.600	0.870	0.033
59. BD (1) C9-H23	92. BD*(1) C6-C9	0.630	1.050	0.023
59. BD (1) C9-H23	99. BD*(1) C9-C14	0.620	1.060	0.023
59. BD (1) C9-H23	102. BD*(1) C10-C14	3.330	1.040	0.053
59. BD (1) C9-H23	109. BD*(1) C14-O22	1.160	0.850	0.028

59. BD (1) C9-H23	203. RY (1) C6	0.660	1.920	0.032
59. BD (1) C9-H23	336. RY (1) C14	0.810	1.650	0.033
60. BD (1) C10-C14	93. BD*(1) C7-C10	1.790	1.170	0.041
60. BD (1) C10-C14	95. BD*(1) C7-O19	3.430	0.980	0.052
60. BD (1) C10-C14	99. BD*(1) C9-C14	2.460	1.180	0.048
60. BD (1) C10-C14	101. BD*(1) C9-H23	2.240	1.030	0.043
60. BD (1) C10-C14	103. BD*(1) C10-H24	1.110	1.030	0.030
60. BD (1) C10-C14	120. BD*(1) O22-H32	1.780	1.010	0.038
60. BD (1) C10-C14	222. RY (3) C7	1.880	2.290	0.058
60. BD (1) C10-C14	252. RY (1) C9	0.960	1.880	0.038
60. BD (1) C10-C14	471. RY (1) O22	0.770	1.270	0.028
61. BD (1) C10-H24	90. BD*(1) C4-C7	3.580	1.010	0.054
61. BD (1) C10-H24	93. BD*(1) C7-C10	0.670	1.050	0.024
61. BD (1) C10-H24	95. BD*(1) C7-O19	1.370	0.860	0.031
61. BD (1) C10-H24	99. BD*(1) C9-C14	3.320	1.060	0.053
61. BD (1) C10-H24	102. BD*(1) C10-C14	0.580	1.040	0.022
61. BD (1) C10-H24	109. BD*(1) C14-O22	1.360	0.840	0.030
61. BD (1) C10-H24	220. RY (1) C7	0.700	1.760	0.031
61. BD (1) C10-H24	337. RY (2) C14	0.750	2.080	0.035
62. BD (1) C11-C12	84. BD*(1) C2-C8	2.460	1.080	0.046
62. BD (1) C11-C12	96. BD*(1) C8-C11	2.720	1.160	0.050
62. BD (1) C11-C12	106. BD*(1) C11-H25	0.940	1.070	0.028
62. BD (1) C11-C12	107. BD*(1) C12-C15	2.810	1.150	0.051
62. BD (1) C11-C12	112. BD*(1) C15-O21	2.600	0.970	0.045
62. BD (1) C11-C12	236. RY (1) C8	2.270	1.970	0.060
62. BD (1) C11-C12	241. RY (6) C8	0.710	2.060	0.034
62. BD (1) C11-C12	303. RY (2) C12	0.680	3.540	0.044
62. BD (1) C11-C12	306. RY (5) C12	0.600	4.440	0.046
62. BD (1) C11-C12	308. RY (7) C12	0.990	6.290	0.071
62. BD (1) C11-C12	310. RY (9) C12	0.760	5.900	0.060
62. BD (1) C11-C12	314. RY (13) C12	0.740	4.080	0.049
62. BD (1) C11-C12	317. RY (16) C12	0.920	2.900	0.046
62. BD (1) C11-C12	353. RY (1) C15	1.260	1.810	0.043
62. BD (1) C11-C12	356. RY (4) C15	0.660	2.230	0.034
62. BD (1) C11-C12	438. RY (2) O20	0.530	1.380	0.024
63. BD (2) C11-C12	98. BD*(2) C8-C16	19.180	0.290	0.067
63. BD (2) C11-C12	111. BD*(2) C15-C18	19.810	0.280	0.067
63. BD (2) C11-C12	243. RY (8) C8	0.590	1.690	0.028
63. BD (2) C11-C12	360. RY (8) C15	1.050	1.290	0.033
63. BD (2) C11-C12	437. RY (1) O20	1.550	0.880	0.033
64. BD (1) C11-H25	84. BD*(1) C2-C8	0.520	0.950	0.020
64. BD (1) C11-H25	96. BD*(1) C8-C11	0.690	1.030	0.024
64. BD (1) C11-H25	97. BD*(1) C8-C16	3.590	1.030	0.054

64. BD (1) C11-H25	104. BD*(1) C11-C12	0.590	1.060	0.022
64. BD (1) C11-H25	107. BD*(1) C12-C15	3.560	1.020	0.054
64. BD (1) C11-H25	108. BD*(1) C12-O20	1.500	0.840	0.032
64. BD (1) C11-H25	236. RY (1) C8	1.390	1.840	0.045
64. BD (1) C11-H25	302. RY (1) C12	0.730	1.680	0.031
64. BD (1) C11-H25	308. RY (7) C12	0.530	6.160	0.051
65. BD (1) C12-C15	104. BD*(1) C11-C12	2.180	1.190	0.046
65. BD (1) C12-C15	106. BD*(1) C11-H25	1.820	1.070	0.039
65. BD (1) C12-C15	110. BD*(1) C15-C18	2.150	1.180	0.045
65. BD (1) C12-C15	116. BD*(1) C18-H28	1.990	1.040	0.041
65. BD (1) C12-C15	118. BD*(1) O20-H30	1.610	1.030	0.036
65. BD (1) C12-C15	119. BD*(1) O21-H31	1.680	1.020	0.037
65. BD (1) C12-C15	286. RY (1) C11	1.320	1.750	0.043
65. BD (1) C12-C15	403. RY (1) C18	1.070	1.780	0.039
66. BD (1) C12-O20	96. BD*(1) C8-C11	1.150	1.350	0.035
66. BD (1) C12-O20	104. BD*(1) C11-C12	0.820	1.380	0.030
66. BD (1) C12-O20	110. BD*(1) C15-C18	1.160	1.360	0.035
66. BD (1) C12-O20	286. RY (1) C11	0.640	1.930	0.031
66. BD (1) C12-O20	353. RY (1) C15	0.650	2.000	0.032
67. BD (1) C14-O22	92. BD*(1) C6-C9	0.860	1.370	0.031
67. BD (1) C14-O22	93. BD*(1) C7-C10	0.960	1.380	0.032
68. BD (1) C15-C18	107. BD*(1) C12-C15	2.520	1.150	0.048
68. BD (1) C15-C18	108. BD*(1) C12-O20	2.670	0.980	0.046
68. BD (1) C15-C18	113. BD*(1) C16-C18	2.150	1.190	0.045
68. BD (1) C15-C18	114. BD*(1) C16-H26	1.860	1.070	0.040
68. BD (1) C15-C18	116. BD*(1) C18-H28	0.650	1.040	0.023
68. BD (1) C15-C18	302. RY (1) C12	1.250	1.820	0.043
68. BD (1) C15-C18	305. RY (4) C12	0.580	2.700	0.035
68. BD (1) C15-C18	370. RY (1) C16	0.990	1.780	0.038
68. BD (1) C15-C18	371. RY (2) C16	1.190	1.740	0.041
69. BD (2) C15-C18	98. BD*(2) C8-C16	21.420	0.290	0.071
69. BD (2) C15-C18	105. BD*(2) C11-C12	17.890	0.290	0.065
69. BD (2) C15-C18	309. RY (8) C12	0.670	0.790	0.020
69. BD (2) C15-C18	372. RY (3) C16	0.990	0.780	0.025
69. BD (2) C15-C18	374. RY (5) C16	0.750	1.810	0.033
69. BD (2) C15-C18	454. RY (1) O21	1.460	0.880	0.032
70. BD (1) C15-O21	104. BD*(1) C11-C12	1.070	1.390	0.034
70. BD (1) C15-O21	110. BD*(1) C15-C18	0.700	1.370	0.028
70. BD (1) C15-O21	113. BD*(1) C16-C18	0.910	1.380	0.032
70. BD (1) C15-O21	302. RY (1) C12	0.680	2.010	0.033
71. BD (1) C16-C18	84. BD*(1) C2-C8	2.810	1.060	0.049
71. BD (1) C16-C18	97. BD*(1) C8-C16	2.480	1.140	0.047
71. BD (1) C16-C18	110. BD*(1) C15-C18	2.040	1.160	0.043

71. BD (1) C16-C18	112. BD*(1) C15-O21	3.540	0.950	0.052
71. BD (1) C16-C18	114. BD*(1) C16-H26	0.960	1.050	0.028
71. BD (1) C16-C18	116. BD*(1) C18-H28	1.110	1.020	0.030
71. BD (1) C16-C18	236. RY (1) C8	1.190	1.950	0.043
71. BD (1) C16-C18	237. RY (2) C8	0.810	2.040	0.036
71. BD (1) C16-C18	354. RY (2) C15	1.860	2.860	0.065
71. BD (1) C16-C18	506. RY (1) H26	0.770	1.420	0.030
71. BD (1) C16-C18	518. RY (1) H28	0.640	1.180	0.025
72. BD (1) C16-H26	84. BD*(1) C2-C8	0.610	0.940	0.021
72. BD (1) C16-H26	96. BD*(1) C8-C11	3.680	1.030	0.055
72. BD (1) C16-H26	97. BD*(1) C8-C16	0.520	1.030	0.021
72. BD (1) C16-H26	110. BD*(1) C15-C18	2.960	1.040	0.050
72. BD (1) C16-H26	113. BD*(1) C16-C18	0.590	1.050	0.022
72. BD (1) C16-H26	116. BD*(1) C18-H28	0.670	0.910	0.022
72. BD (1) C16-H26	236. RY (1) C8	1.220	1.840	0.042
72. BD (1) C16-H26	242. RY (7) C8	0.510	2.710	0.033
72. BD (1) C16-H26	403. RY (1) C18	0.800	1.650	0.032
73. BD (1) O17-H27	79. BD*(1) C1-C2	4.970	1.260	0.071
73. BD (1) O17-H27	81. BD*(1) C1-C3	1.120	1.110	0.032
73. BD (1) O17-H27	121. RY (1) C1	1.570	1.810	0.047
73. BD (1) O17-H27	122. RY (2) C1	0.780	2.330	0.038
73. BD (1) O17-H27	125. RY (5) C1	0.560	2.670	0.035
74. BD (1) C18-H28	97. BD*(1) C8-C16	3.090	1.040	0.051
74. BD (1) C18-H28	107. BD*(1) C12-C15	3.240	1.030	0.052
74. BD (1) C18-H28	112. BD*(1) C15-O21	1.260	0.850	0.029
74. BD (1) C18-H28	113. BD*(1) C16-C18	0.680	1.060	0.024
74. BD (1) C18-H28	114. BD*(1) C16-H26	0.630	0.950	0.022
74. BD (1) C18-H28	353. RY (1) C15	0.700	1.690	0.031
74. BD (1) C18-H28	370. RY (1) C16	1.050	1.660	0.037
75. BD (1) O19-H29	90. BD*(1) C4-C7	4.360	1.200	0.065
75. BD (1) O19-H29	220. RY (1) C7	1.710	1.960	0.052
75. BD (1) O19-H29	224. RY (5) C7	0.530	2.830	0.034
75. BD (1) O19-H29	270. RY (2) C10	0.650	1.650	0.029
76. BD (1) O20-H30	107. BD*(1) C12-C15	3.900	1.220	0.062
76. BD (1) O20-H30	302. RY (1) C12	1.770	1.890	0.052
77. BD (1) O21-H31	107. BD*(1) C12-C15	3.860	1.230	0.061
77. BD (1) O21-H31	353. RY (1) C15	1.880	1.900	0.053
78. BD (1) O22-H32	102. BD*(1) C10-C14	4.050	1.250	0.064
78. BD (1) O22-H32	253. RY (2) C9	0.560	1.800	0.028
78. BD (1) O22-H32	336. RY (1) C14	1.980	1.860	0.054
78. BD (1) O22-H32	337. RY (2) C14	0.540	2.300	0.032

Table S4. Interactions between Que and oxidative endogenous enzymes identified during the MD studies.

NOX			XO			MPO			LOX			MAO-A		
Residue	Interaction	%	Residue	Interaction	%	Residue	Interaction	%	Residue	Interaction	%	Residue	Interaction	%
Ser 115	H-bond	74	Ala 1079	H-bond	40	Glu 116	H-bond	19	Arg 101	Pi-cation	69	Thr 336	H-bond	96
Cys 133	H-bond	54	Glu 879	Water bridge	38				Tyr 142	Water bridge	41	Asn 181	Water bridge	74
Leu 241	H-bond	43	Pro 1076	Water bridge	33				Arg 101	Pi-cation	35	Cys 323	H-bond	70
Phe 245	Pi-pi stacking	40	Glu 802	Water bridge	29				Gln 141	H-bond	32	Phe 208	Pi-pi stacking	68
Cys 242	H-bond	32	Glu 802	Water bridge	24				Glu 134	Water bridge	32	Tyr 444	Water bridge	68
Phe 245	Pi-pi stacking	31	Asn 768	H-bond	23				Val 110	H-bond	31	Tyr 407	Water bridge	40
Lys 134	Pi-cation	30	Glu 802	H-bond	21				Val 110	Water bridge	21	Ala 111	Water bridge	35
Gly 158	H-bond	27	Phe 1009	Pi-pi stacking	16				Arg 138	Water bridge	18	Phe 208	H-bond	34
Asp 138	Water bridge	26	Phe 1009	Pi-pi stacking	10				Val 110	Water bridge	18	Ala 111	Water bridge	25
Pro 298	H-bond	19	Mos 1328	H-bond	10				Arg 101	Water bridge	17	Gln 215	Water bridge	19
Asn 135	Water bridge	15							Asp 166	Water bridge	16	Phe 208	Water bridge	11
Tyr 188	Water bridge	15							Tyr 142	Water bridge	15	Asn 181	Water bridge	11
Phe 245	Water bridge	14							Arg 68	Water bridge	10			
Gly 158	H-bond	14							Arg 112	Water bridge	10			
Cys 133	H-bond	13												
Tyr 188	Water bridge	12												
Cys 242	H-bond	10												
Gly 158	Water bridge	10												
